# Transnational pharmacogovernance: emergent patterns in the jazz of pharmaceutical policy convergence

**DOI:** 10.1186/s12992-018-0402-5

**Published:** 2018-08-22

**Authors:** Mary Wiktorowicz, Kathy Moscou, Joel Lexchin

**Affiliations:** 1 School of Health Policy and Management, York University, Toronto, Canada; 2 WHO Collaborating Centre for Governance, Accountability and Transparency in the Pharmaceutical Sector, University of Toronto, Toronto, Canada; 3 Faculty of Education, Brandon University, Brandon, Canada; 4 University Health Network, Faculty of Medicine, University of Toronto, Toronto, Canada

**Keywords:** Pharmaceutical regulation, Medicines policy, Transnational governance, Transnational regulation, Pharmacogovernance, Policy network, Policy convergence, Drug safety, Pharmacovigilance

## Abstract

**Background:**

As a transnational policy network, the International Council for Harmonization of Technical Requirements for Registration of Pharmaceuticals for Human Use (ICH) aligns international regulatory standards to address the pressures of globalization on the pharmaceutical industry and increase access to new medicines. Founding ICH members include regulators and pharmaceutical industry trade associations in the European Union, the United States and Japan. In this paper we explore the manner in which state interdependence fosters the conditions for regulatory harmonization by tracing the underlying parallels between ICH and member state pharmacogovernance to clarify emergent patterns in regulatory policy convergence.

**Results:**

A shift to the life cycle approach to pharmaceutical regulation corresponded with international convergence in pre-market standards as emphasis shifted to post-market standards where convergence remains unresolved. Transnational pharmacogovernance was found to concentrate regulatory authority within a co-regulatory model of bilateral negotiation with pharmaceutical trade associations in defining safety and efficacy standards. Given a context of state interdependence, parallels were found between transnational and ICH member pharmacogovernance modes that guide policy development. Divergent modes of state regulatory governance that re-calibrate perceptions of risk and risk mitigation were found to coincide with post-market policy dissonance.

**Conclusion:**

Although interdependence fostered harmonization in pre-market standards and aligned with increased focus on post-market approaches, the confluence of divergent state governance modes and perceptions of risk may inspire improvisation in post-market standards. As the ICH expands to an ensemble with a greater global reach, further research is needed to clarify the manner in which interdependence shapes transnational pharmacogovernance and the conditions that foster policy convergence in the public interest.

**Electronic supplementary material:**

The online version of this article (10.1186/s12992-018-0402-5) contains supplementary material, which is available to authorized users.

## Background

Policy problems whose resolution is most readily addressed within a global context present new governance challenges. Complexities arise as states shaped by different political, economic and cultural traditions seek to harmonize regulatory standards [1, 2]. While globalization and the ascendance of capital markets have been found to hasten policy convergence, the precise causal links between the two remain underdeveloped [3]. Although case studies exploring the links between globalization and policy convergence may cast states as independent actors with the capacity to assume completely different policy instruments, the approach neglects their economic interdependence, highlighting the need for more nuanced analyses exploring the underlying strategic political dimension [4, 5]. Interdependence reflects the mutual dependency of state interests, where the national policy of one state affects those of others. Factors that affect interdependence include the *symmetry* of states’ economic power and influence, the *type* of political strategy that states pursue, and the *degree* to which one state depends on others [6].

Despite the underlying interdependence, state agency as manifested through domestic institutions that influence the allocation of authority, remains an important thread, as policy preferences are often artifacts of their institutional context [7, 8]. Alternate state governance orientations and the institutional constraints and opportunities they present to actors involved in the policy process affect behavior [9], and have been found to foster divergent healthcare [10, 11], chemicals [12, 13] and pharmaceutical policies [1, 14, 15]. As regulatory dissonance can create trade barriers for multinational companies, states share the burden of harmonizing their regulatory standards to forge a path for international trade and increase citizens’ access to new medicines. Even though only one in nine new drugs offer a significant therapeutic advance over current medicines [16], market interdependence nonetheless exerts pressures on states to pursue harmonization [17, 18]. Medicines regulation is a case rich in insights that shed light on how transnational networks allocate authority among state and societal actors in developing global standards that balance societal risks with the benefits of a competitive domestic industry [15, 19]. In the pharmaceuticals sector, a transnational public-private policy network, the International Council for Harmonization of Technical Requirements for Registration of Pharmaceuticals for Human Use (ICH) was formed to harmonize international regulatory standards to address the concerns of the pharmaceutical industry that different international regulatory criteria created trade barriers and increased drug development costs. Streamlined harmonized standards were further sought to reduce lengthy product reviews [17]. Originally comprised of the regulators and industry associations in the European Union (EU), Japan and the United States (US), the ICH fosters transnational cooperation in order to align regulatory standards and reduce trade barriers in response to globalization [20]. Membership was expanded in 2016 to include Canada, Switzerland, Brazil, South Korea and China, while Australia and Taiwan are observers; the first step toward membership.

Understanding how transnational networks’ delegated authority supplants state agency authority is crucial in a context in which global regulatory standards are set by public-private policy networks [21–23]. As regulatory standard setting shifts from a solely national to a transnational concern, where the state no longer holds a monopoly in the policy process, the manner in which medicines’ benefits and harms imbued with scientific uncertainty are addressed through transnational pharmacogovernance is important to understand. Pharmacogovernance is defined as the manner in which governing structures, policy instruments and institutional authority that enable the development, implementation and enforcement of regulatory policies are managed to promote societal interests including protection of public safety [24]. The objective of this paper is to compare the pharmacogovernance framework guiding the ICH to that of six member jurisdictions (European Union, United Kingdom, France, United States, Canada and Japan) with established regulatory frameworks to allow generalizability, yet sufficient range to cover a spectrum of approaches.

### Theoretical lens

In network governance theory, governments rely on transnational networks comprised of policy actors outside their hierarchical control to negotiate global norms and rules of engagement, through which diverse resources are mobilized [25–27]. As transnational networks become adept at responding to emerging political challenges, they contribute to a multinucleated global system comprised of a series of sectoral networks that create circuits of power organized around issue areas [23, 28]. States’ concern for their potential loss of sovereignty, given transnational networks’ increasing influence, is countered by the economic benefits of harmonized, streamlined regulatory processes and common standards that reduce the necessity to conduct randomized controlled trials for different countries, diminish company costs and shift resources to developing new medicines. In theory, the lower product development costs would lead to greater investment in research and development. States’ interest in expanding market access combined with a view of regulation as a technical non-discretionary matter, lends legitimacy to the transnational public-private partnership network model [29]. Regulatory networks that involve government and private sector actors, referred to as public-private policy networks, institutionalize cooperative relationships, and create a forum where public actors mediate negotiations with private actors to shape international policy. In private interest governance, private associations develop public policies with oversight from public authorities [30].

As globalization advances, the ways in which international institutions shape and are shaped by domestic institutional politics becomes more difficult to understand [31]. An interdependence lens that incorporates historical institutionalism offers a means to assess the role of the ICH as a global governance network that mediates transnational regulatory harmonization [32]. In this paper, we analyze how ICH member states’ pursuit of harmonized standards, through transnational cooperation, affects the development of global standards by comparing the nature, sequencing and unfolding of state, supranational and transnational pharmacogovernance processes. In exploring the relationship between interdependence and power, where power is understood as domestic institutional capacity to articulate a set of rules that shape harmonized standards [31], we address the question of how different institutional configurations affect the expression of harmonized rules and global norms that guide regulatory approaches. In exploring the political context that informs transnational regulatory approaches, we consider the question of which jurisdictions determine the terms of interdependence, the coalitions that form and whom they advantage [4].

Guided by the lens of the new interdependence, we trace parallels between supranational and state governance processes to elucidate emergent patterns and the manner in which the interactions between them may influence global politics and domestic institutions [31]. State and supranational pharmacogovernance approaches in Europe, North America and Japan were analyzed to clarify the emergent patterns and parallels to transnational regulatory governance.

The concept of conversion, in which different state governance modes can introduce new actors whose goals may alter an institution’s objectives and lead to policy discordance, is incorporated in the analysis [33]. We identify how transnational alliances introduce different sets of actors that lead to the emergence of new conceptual approaches at the transnational level. Where such approaches may not align with domestic policy, the conceptual discordance between transnational and domestic actors reflects regulatory layers that can destabilize institutions and re-shape global politics [33, 34]. After describing the methods, the sections that follow consider supranational and state regulatory governance and trace the parallels between them to clarify the manner in which interdependence among ICH members predisposes them toward policy convergence [35].

## Methods

Pharmacogovernance guiding the ICH was compared to that of six member jurisdictions including the European Union, United Kingdom, France, United States, Canada and Japan given their relative similarity as developed nations and regions with established regulatory frameworks to allow generalizability, yet sufficient range to cover a spectrum of regulatory approaches [36]. The research involved a review of literature, policy and technical reports (1990–2017) along with 26 semi-structured interviews with key informants in international regulatory agencies and related organizations in 2007, 2010, 2015–2017. Interviewees were engaged in pharmaceutical regulatory and policy spheres including the ICH Secretariat, the European Medicines Agency (EMA), an EMA consumer representative, US Food and Drug Administration (FDA), US Veterans Administration Center for Medication Safety, US Developing Evidence to Inform Decisions about Effectiveness research network, UK Medicines and Healthcare products Regulatory Agency (MHRA), UK National Institute for Health and Clinical Excellence (NICE), UK Drug Safety Research Unit, Haute Authorité de Santé in France, a regional pharmacovigilance center in France, an editor of *La revue Prescrire* the independent French drug bulletin, and Canadian provincial drug plan representatives. The FDA and Health Canada also offered written responses to interview questions. Ethics approval was attained from York University ethics certificate e2015–141.

Interview schema were informed by a review of the literature, agency administrative and policy documents, publicly available government documents, newspaper articles, and authors knowledge of regulatory policy. The documents containing discourse pertaining to ICH and state pharmacogovernance were read iteratively to illuminate areas for further exploration. The interview guide that was developed was modified for different groups of interviewees based on their role in drug regulation. The interview guides were continuously updated to probe additional relevant information that was uncovered during preceding key informant interviews. Although the schema was not pilot tested it was modified based on initial interviews to clarify the intent of questions.

Content analysis of transcribed interviews was conducted using Atlas.ti by two authors (KM and MW) who independently and collaboratively analyzed and organized the data into emergent themes through email discussions [37]. Data were coded and analyzed using an analytic framework. Framework domains were established a priori. A codebook (Additional file 1) was created with operational definitions for each framework domain, with illustrative examples, to guide the independent analysis by the study authors. Information from the interviews and document analysis were cross-referenced to ensure consistency between the two in order to characterize patterns in international regulatory governance and policy approaches. When there were areas of inconsistency we contacted the interviewees to confirm our interpretation of what they said, conducted additional interviews and identified additional confirmatory documentation. Key informant quotes that succinctly characterized an emergent pattern within a theme were included in the codebook to offer illustrative examples.

To elucidate patterns in state and transnational institutional governance across jurisdictions over time and clarify how regulators interact at the global level to shape transnational governance processes [38], we developed a framework to compare their regulatory authority, state-societal relations, representation and the role of legal channels [29, 39]. A comparative analysis of distinct state governance modes and approaches to risk management enabled us to clarify the conditions that foster improvisation in post-market regulatory policy, the coalitions interdependence supports, who they advantage and how they are leveraged transnationally to foster policy convergence.

## Results: International approaches to pharmacogovernance

### Transnational network governance - *Co-decision-making*

As a transnational network, the ICH is a governance forum whose decisions culminate in a set of harmonized regulatory standards to which its members agree.^1^ The ICH began as biennial meetings of regulators and industry associations in the EU, US and Japan in 1990, who solidified their pursuit of harmonization in 2003. The ICH secretariat was initially funded by and housed in the International Federation of Pharmaceutical Manufacturers & Associations head office in Geneva. Governance occurred through the ICH Steering Committee where each regulator and industry association was represented by two members, who determined areas for harmonization and set global standards, such as the Common Technical Document for new product dossier submissions that reduced duplicate testing.

In the first 25 years, regulators and trade association representatives *co-decided* the standards for market entry of new drugs. Criticism that pharmaceutical industry representatives chaired committees setting regulatory standards prompted the ICH to amend its governance in 2012, to confine the committee chair to a regulatory member [40]. As regulators were under increased scrutiny over their interdependence with industry, further ICH governance reforms in 2015 clarified the leading role of regulators compared to that of industry and expanded international membership [41]. Prior to the 2015 governance reforms, industry association members could propose areas for harmonized standards. If consensus could not be attained, industry representatives and regulators, who held equal numbers of votes, would vote to accept or decline a guideline.

The reforms replaced the Steering Committee with an Assembly that identifies and approves areas for standards development and a Management Committee that oversees operational matters. Regulatory authorities and industry hold equal seats in the Assembly and Management Committee where decisions are made through consensus. In exceptional cases without consensus, votes are taken and regulators make the final decision. The reforms also shifted industry funding of the ICH to membership fees.

**Fig. 1:**
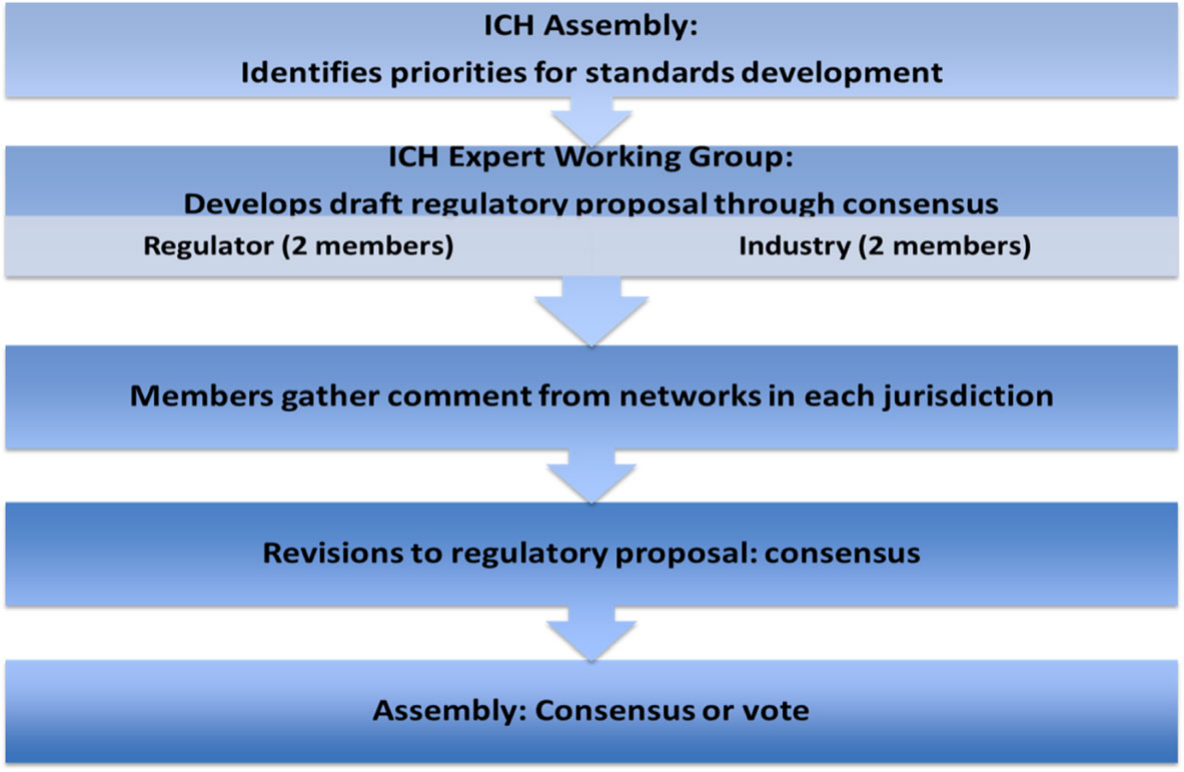
Development of Harmonized Regulatory Standards

*Co-decision-making* in drafting rules also occurs through Expert Working Groups where standards are formed and consensus sought [42]. The justification given for equal representation of industry and regulators is industry’s technical expertise. Industry is primarily involved in the initial stages in developing a ‘technical document’ that includes statements of the scientific discussions in the working group. Once a guideline is drafted, comment is gathered sequentially from industry across jurisdictions and then from regulator networks, with industry members gathering the first set of comments (Fig. 1).^2^ It is then up to regulators to transform this technical document with or without changes, into a guideline for consideration by the Management Committee and the Assembly. The ICH addresses transparency by posting its draft guidelines on its website and allows comments beyond member organizations. A third party would at the same time need to be aware of the window for comment. After considering comments, and the process of consensus and approval in the Assembly, the guideline becomes a global standard, that regulatory members commit to implement (Fig. 1, Step 4). A central feature is conversion of the ‘soft power’ of the ICH to attain consensus on common goals and technical guides into the ‘hard power’ of state policy and legislation [43].

As a transnational pharmacogovernance regime, the ICH legitimizes pharmaceutical associations’ negotiation and argumentative persuasion in the development of standards that guide public policy [42, 44, 45]. The ICH centralizes the development and transfer of standards, introducing a concentrated global authority parallel to the transnational authority of the EMA within the European Commission [46]. As the ICH is a global norms initiator, standards are adopted beyond the three founding jurisdictions including Brazil, Singapore, South Africa, Mexico and Ghana.

### State regulatory pharmacogovernance – *Interdependent and independent decision-making*

Interdependence influences network policy decisions to address the governance of risks that are multijurisdictional, complex and/or ambiguous, such as the risks posed by pharmaceuticals [47–49]. Although the intent of the ICH was to harmonize national regulatory standards, the shift to a life-cycle approach to drug assessment fostered policy convergence among ICH members in pre-market standards but left unresolved dissonance in post-market approaches (Table 1) [50]. The life-cycle approach to regulation acknowledges that some types of information about a drug are not available prior to marketing (including rare adverse events, interactions with other drugs, long-term effects and effects on patient groups excluded from clinical trials), giving regulators a longer period to gather evidence.

**Table 1:** Convergence in International Medicines Regulatory Standards

	ICH	EU	Canada	Japan	US	
		EMA Britain France			Pre-2013	Post-2013
Pre-market standards						
Rodent carcinogenicity tests: for medicines used for 3 or 6 months^a^	6 months	6 months	6 months	6 months	3 months	6 months
Chronic toxicity tests in animals^b^	6 months	6 months	6 months (pre-ICH 18 months)	6 months (pre-ICH 12 months)	12 months	9 months^e^
Length of RCTs for medicines used for chronic conditions^a^	6 months	6 months	6 months	6 months	12 months	Indication-specific
Timing of toxicity tests^c^	After initially taking medicine	After initially taking medicine	After initially taking medicine	In accordance with the drug characteristics	At steady state	Determined by the clinical development plan^f^
Expedited authorization	–	Adaptive pathways	Conditional approval for life threatening conditions	Expedited approval for regenerative therapies (stem cells, gene therapy)	Fast-track for life threatening conditions	Expedited approval for serious conditions
Post-market standards						
ADR Reporting^d^	If suspect ADR relates to a medicine	If suspect ADR relates to a medicine	If suspect ADR relates to a medicine	If suspect ADR relates to a medicine	All ADRs regardless of suspected relationship to a medicine	All ADRs regardless of suspected relationship to a medicine
Phamacovigilance	Risk Management Plan	Risk Management Plan	Risk Management Plan	Risk Management Plan; Early post-market phase vigilance; Good post-market study practice; Re-approval^g^	Risk Evaluation & Mitigation; Commissioned Sentinel data mining	Risk Evaluation & Mitigation; Commissioned Sentinel data mining

^a^Abraham and Reed [46]

^b^Abraham and Reed [47]

^c^Yu, Bischoff and Tweedie [48]

^d^Castle and Kelly [49]; Kesselheim et al. [50]

^e^In certain cases, non-rodent studies of up to 6 months can be appropriate in Japan and the US [51]. Shorter non-rodent toxicity studies are for example allowed when immunogenicity or intolerance confounds conduct of longer term studies; in cases of repeated short-term drug exposure even if clinical trial duration exceeds 6 months; for drugs administered on a chronic basis to reduce the risk of recurrence of cancer; and for drugs for indications for which life expectancy is short

^f^FDA written responses to interview questions [51]

^g^Faden and Milne

Even in the pre-market phase, state-level implementation varies reflecting independence in standards for reporting adverse effects as evidenced by the FDA use of a different periodic safety update reporting (PSUR) standard. Companies must adhere to the FDA Guidance to Industry, or apply for a waiver to use the ICH/EMA guideline even though the FDA Guidance to Industry is retired upon publication of the ICH guidance in the Federal Register [50]. In addition, the ICH and the Council for International Organizations of Medical Sciences (CIOMS) require pharmaceutical companies to report adverse effects with a possible causal relationship to a drug. In the US, sponsors must report all adverse events irrespective of the likelihood of a causal relationship, reflecting American independence.

*“An adverse event as we define it does not have to have a suspicion of causality whereas the ICH standard says only if suspected [it] is related to a medicine. We think that casting it broadly like that is the best approach for seeing things that maybe you might not see otherwise”* (US4-FDA, 2015).

Divergence in these standards creates situations in which adverse drug event profiles that guide market authorization decisions, can differ among international regulators [51]. Despite their interdependence within the transnational network, states thus retain autonomy to adapt ICH guidelines [50, 52]. Members’ alternate perceptions of pharmaceutical risks and approaches to address them are reflected in discordant state regulatory standards and responses to safety issues (see Table 1) [51, 53, 54].^3^ In Japan for example, clinical trial bridging studies are required that include Japanese patients [55]. Overall, American and Japanese standards differ most from those of the ICH and Europe reflecting greater independent decision making [56, 57]. Even when the FDA aligns its policies with ICH standards, it does so with qualifications [50, 51, 58].

State *interdependence* is alternatively reflected in the development of a new ICH pharmacovigilance guideline that was designed to address uncertainties in post-market safety, that extended the original ICH role of harmonization. In response to political pressure from the European Parliament for more effective post-market surveillance (following the withdrawal of cerivastatin (Baycol®) in 2001 and rofecoxib (Vioxx®) in 2004), the EMA sought a new approach. Existing PSURs capture less than 10 % of adverse drug events, limiting knowledge about drug benefit to harm ratios. The European Commissioner for Industry insisted the EMA enhance pharmacovigilance. Ministers of Health in the EU also directed their national medicines regulators to develop pharmacovigilance plans [59]. The EMA and EU Heads of Regulatory Agencies group formed a committee to develop a risk management approach that resembled the UK Medicines Control Agency’s risk management plan; the two risk management plans (it) then merged.^4^ British Medicines Control Agency regulators Waller and Evans emphasized collaboration with academic clinical pharmacologists and epidemiologists to advance post-market analytic methods. They advised multi-center epidemiologic studies focused on serious or unexpected adverse drug events and introduced the term ‘pharmacovigilance specification’ that refers to a safety specification and pharmacovigilance plan that guided the ICH pharmacovigilance plan (E2E) and the EMA’s risk management plan [60].

The network overseeing the development of the risk management plan commenced within CIOMS, a World Health Organization and UNESCO sponsored committee comprised of regulators and pharmaceutical industry representatives, that develops regulatory safety standards including the practice of pharmacovigilance. CIOMS became a pre-ICH consultation forum in which priorities for harmonization were discussed before their presentation in the ICH. The concept of the pharmacovigilance plan was introduced to the ICH Safety Working Group by two members of CIOMS, a British regulator and an industry representative, and became an ICH standard 2 years later [61].

The essence of Waller and Evan’s advice would appear to have been lost in translation however. In the EMA standard, the pharmaceutical company develops and oversees the risk management plan, approved by the regulator [59]. Pharmacoepidemiologists found the ICH introduced the risk management plan prematurely, before it was tested [62]. Giezen et al. [63] found weaknesses in risk management plans that affected their effectiveness. Conflict of interest may arise when post-market safety studies are designed by the drug sponsor, who may be reluctant to pursue the rigorous research needed to establish causality for adverse events that could jeopardize market share [64–66]. Rather than focus primarily on the science of risk reduction, risk management involved balancing inter-related risks such as the risk to patients should a drug remain on the market and risk to the reputation of the regulatory agency and the pharmaceutical company should a drug be withdrawn [67]. Without evidence of causality, the sponsor could justify non-reporting.

*“I mean there’s lots of evidence that manufacturer produced information is biased…but then we rely overwhelmingly on manufacturers’ information anyway in all these decisions”* (UK5-NICE 2010).

Concern was expressed by European key informants that the process used to develop risk management plans leads regulators to validate a pharmacovigilance strategy with inherent flaws.

*“They don’t actually require manufacturers to go out and establish what is happening with these drugs, and what manufacturers imagine is happening with these drugs I’m sure can be miles away from what patients experience. And secondly, it is being left to the manufacturers themselves to produce and analyze and present these data. And those seem to be two very fatal flaws. It doesn’t matter if they work within their limitations, but what I object to, is a regulatory system which tells the public how trustworthy and competent they are, on the one hand, and have such flimsy processes for establishing benefit/harm ratios”* (UK3-NGO 2007).

Academics in the European Society of Pharmacovigilance found that in the ICH’s orchestration of an approach to pharmacovigilance, scientists were superseded by conference organizers and drug companies. ICH governance excluded the perspectives of academic pharmacoepidemiologists from decision-making concerning risk management plans. The drive for consensus tended to dominate ICH planning rather than in-depth scientific consideration that could have included a pilot test phase of the risk management plan to better understand its impact on public health [62]. Rather than expand the range of possible approaches to assess post-market drug risk, interdependence would appear to have limited the solution set given the narrow representation within the ICH.

*“A flaw is that we depend on the companies to carry out the (post-market*) *studies. We have tried to put in the maximum of protection. It would be much better to have a system in which academics or HAS carried out the study; the financing could still come from the industry but this would permit independence, and alleviate current doubts”* (France3-Haute Authorité de Santé 2007).

The American and Japanese approach to pharmacovigilance alternatively reflects *independence.* In response to a US Government Accountability Office report [68] that found the FDA placed a heavy reliance on drug sponsors to inform it of safety issues, rather than independently seeking the information, the FDA Amendments Act (FDAAA) requires the FDA to engage in *active* surveillance independent of product sponsors by contracting independent research centers to investigate safety signals [69] and increased the FDA’s resources and authority to do so [67]. While the EMA relies on product sponsors to conduct post-market research, the FDA also independently commissions researchers to mine electronic healthcare databases and conduct epidemiologic analyses to identify drug safety signals independent of product sponsors.

*“Occasionally they’ll say that they thought it was [n’t] related to anything. Companies think it’s about causality determination as well. Of course, for things we’re interested in, we can do our own causality assessment”* (US4-FDA, 2015).

The FDA still requires companies to conduct post-market studies. While the FDAAA granted the FDA power to fine companies that do not complete the post-market studies to which they agree, the FDA has never used this power [70].^5^

The FDA’s cooperative agreements with the Centers for Medicare & Medicaid Services and the Veterans Administration expand its research expertise and access to databases. *“The FDA needs these questions answered and we can’t do it ourselves, so we do it in collaboration with outside groups that have both the data and the expertise”* (US2-FDA, 2010). Active surveillance of healthcare databases is conducted through the Sentinel System to inform FDA decisions. Although the Sentinel System has been criticized for not realizing its vision [71], it allows independent assessment of the effectiveness of FDA safety advisories [72]. Overall, the US approach to *active* pharmacovigilance is distinctive in that it does not rely exclusively on industrial sponsors, revealing its independence (Table 1).

*“Our regulations don’t contemplate…or require a pharmacovigilance plan so we think the guideline is a good guideline it’s just that we don’t have a regulatory mechanism for it”* (US4-FDA, 2015).

Canada adopted risk management plans in its progressive licensing framework [73]. The principles that Health Canada enunciated behind progressive licensing are promising but there is no commitment to balance the funding or number of personnel devoted to premarket (75–80%) versus post-market evaluation (20–25%) [74]. In Japan the risk management plan supplements two additional phased approaches to post-market assessment: re-examination that requires sponsors to collect post-market data, and a re-evaluation and re-approval system. The Pharmaceutical Affairs Law (2002) changed safety and post-market surveillance by instituting Good Vigilance Practice (GVP) including Early Postmarketing Phase Vigilance (EPPV) to address the low rate of spontaneous reports. Hospitals and physicians complete surveys within 6-months following the launch of new drugs to closely monitor serious adverse drug reactions in accordance with EPPV and GVP. A *post-marketing* survey is not required when a risk management plan is in place. A Good Postmarketing Study Practice (GPSP) standard specifies the studies and surveillance the sponsor must conduct, which the regulator examines 4, 6 or 10 years (depending on product category) after product launch [75]. The Pharmaceuticals and Medical Devices Agency (PMDA) will also mine healthcare databases to uncover signals of adverse drug events [76].

In Japan, the regulator’s product safety assessments are reviewed by an independent advisory body to the Ministry of Health, Labour and Welfare, the Pharmaceutical Affairs and Food Sanitation Council (PAFSC).^6^ The Minister may request a product be re-evaluated at any time based on the advice of the PAFSC whose reviews include adverse drug reaction and GPSP reports. Requiring *re-approval* incents sponsors to complete GPSP studies. When the characteristics of a drug calls for intensive investigation, the MHLW selects medical institutions to conduct an early post-marketing phase safety survey [75, 77]. The range of tools Japan uses through GVP and GPSP form a continuum of pharmacovigilance activities [56]. The Ministry of Health, Welfare and Labour committees on “Judgement of Sufferers from ADRs and Infections” also inform regulatory policy [75].

Given the emphasis on post-market assessment, American and Japanese pre-market standards converge, while post-market standards diverge from the ICH (Table 1). American and Japanese approaches of independent post-market evaluation, that supplement drug sponsors’ post-market study commitments, reflect policy dissonance. The next section compares the parallels between global (ICH), supranational (EU) and national (UK, France, US, Canada and Japan) modes of regulatory governance).

### Tracing parallels between state and transnational modes of pharmacogovernance

The economic interdependence that globalization fosters influences the manner in which state and transnational regulatory network governance evolves [8, 11, 78] (Table 2). As jurisdictions seek to replicate their domestic rule structures at the global level to alleviate their need for institutional change, we assess the manner in which interdependence shapes the transnational network (ICH), and member states (US, UK, France, Japan and Canada) and regions (EU) using a comparative analysis to trace parallels among their approaches to pharmacogovernance [34].

**Table 2:** Modes of Pharmacogovernance

	ICH	EU (UK, France)	Canada	Japan	US
Regulatory authority	Concentrated	Concentrated	Concentrated	Fragmented	Fragmented
State-societal relations	Negotiation	Negotiation	Accommodation	Negotiation/ Managerial Discretion	Managerial Discretion
Representation	Narrow	Narrow	Narrow	Narrow/ Diverse	Diverse
Litigation	None	Limited	Limited	Limited	Extensive: Class Action, Fraud
Decision-making	Cybernetic	Cybernetic	Cybernetic	Analytic	Analytic
Network Governance	Interdependent/co-regulatory decision-making	Interdependent/co-regulatory decision-making	Interdependent	Independent	Independent

#### Regulatory authority

State regulatory governance approaches are guided by different principles of authority that reflect their autonomy to develop and implement policy. Legislative oversight of a regulatory agency can lead to frequent hearings, amendments and special investigations, fragmenting authority [12]*.* The independent authority of regulatory agencies tends to be respected in Europe and Canada that endows their regulators with considerable discretionary power that tends to *centralize* agency authority. Public scrutiny is limited, although not absent as the European ombudsman criticized the EMA for refusing to release unpublished clinical trial reports [79]. European and Canadian regulators possess considerable flexibility in their regulatory judgements and are less likely to be subject to public scrutiny by legislative oversight, judicial review or health advocacy groups via freedom of information laws [80].

In the EU, the EMA creates consistent standards for product authorizations across Member States in the *centralized* process. In harmonizing standards, the EMA balances oversight of pharmaceuticals with a market-supporting regulatory system. The EMA coordinates Member State regulatory agencies responsible for market authorization and post-authorization surveillance through a *decentralized* process and oversees the scientific assessment of new biotechnology products through a *centralized* process. As members of the EMA’s Committee for Human Medicinal Products that oversee EU *centralized* product reviews are seconded from European state regulators, EU level politicians tend to refrain from imposing their agenda [81]. In the *decentralized* process, sponsors may choose the regulator with the least onerous standards, with the market authorization accepted in other Member States through mutual recognition. The European Commission is ultimately responsible for the approval and management of medicines market authorizations through the *centralized* process, whereas Member States retain responsibility through the *decentralized* process [82].

In contrast, the FDA’s authority is *fragmented*. The government’s executive branch sets the terms for agencies such as the FDA to fulfill their responsibilities, including agreements with international counterparts [83]. Congressional oversight through a process of legislative hearings and investigations by special oversight committees constrain the FDA’s discretion and authority [68]. In Japan, regulatory authority is also fragmented through oversight of its regulator, the PMDA, by the PAFSC which provides independent advice to the Minister concerning regulatory standards and the pre- and post-market regulatory reviews undertaken by the PMDA (Table 2).

As a transnational network, the ICH shapes the global regulatory agenda; by concentrating network accountability to a subset of regulators it reconstitutes global authority for developing regulatory standards to a public-private partnership that operates beyond the purview of legislative oversight [84].

#### State-societal relations

Medicines regulators engage in consultations with and endorse industry self-regulation to varying extents. Consultation occurs through regulatory networks that involve government and private sector actors, referred to as public-private policy networks that institutionalize cooperative relationships [30] and create a forum to shape public policy, where public actors mediate negotiations with private actors. In the EU, strategies supporting regulatory governance include ad-hoc consultation bodies and *co-regulation* involving cooperative public-private partnership governance arrangements that develop norms and rules through joint decision-making [30, 84, 85]. Positioned as experts who guide the formation of regulations, pharmaceutical trade associations benefit from a stable regulatory environment that reinforces the mutual dependence between the agency and industry [28]. In the UK and France, pharmaceutical trade associations routinely interact with government; conflicting objectives are resolved through continuous political bargaining, a regulatory model based on a neo-corporatist tradition of negotiation and accommodation with industry through formal relations on a range of policies [86]. “PPPs [Public Private Partnerships] with co-regulation activities characterize corporatist arrangements” [7]. Public and private actors are cast as legitimate partners at the negotiating table allowing industry to influence aspects of the regulatory process that creates a level of government-industry interdependence. The modest resources initially assigned to medicines regulators in France and the UK suggest the model may have been necessary to gain industry compliance [15].

In France, the regulator was guided by a formal administrative framework that operated through informal regulator-industry cooperation whereby industry self-assessed its compliance with the regulator’s safety standards. A *decentralized* style of industry self-regulation evolved in which companies hired an ‘expert’ to assess their compliance with safety standards as the French regulator was inadequately resourced, lacking the technical expertise to ensure implementation of regulations and the ability to act independently. This approach reinforced the agency’s relationship of mutual dependence with the industry association [87].

The French Inspection Générale des Affaires Sociale found institutionalized cooperation with the pharmaceutical industry led to delayed decisions, as reflected in the regulatory agency’s delay in withdrawing benfluorex (Mediator®) from the market. The Inspection Générale des Affaires Sociale report led the French Health Ministry to create a new medicines agency with rules concerning relationships between drug makers and healthcare experts [88]. Industry was not engaged in product reviews in the UK to the extent it was in France, where regulation was considered passive and largely delegated to the industry association [89]. At the same time, the British regulator developed a close working relationship with the Association of the British Pharmaceutical Industry (ABPI) based on negotiation and voluntary cooperation to ensure its members followed regulatory standards [90].^7^ A UK House of Commons Health Committee Report (2005) found the MHRA’s close relationship with industry was reflected in routine consultation on common policy objectives and agreed processes. The Committee advised a fundamental review of the MHRA and recommended improved post-market surveillance of medicines.^8^

The *neo-corporatist* governance approach found in Europe entails state-industry negotiation, an approach the European Commission endorses [85]. Although the EMA supported the establishment of the European Network of Centres for Pharmacoepidemiology and Pharmacovigilance (ENCePP), it lacks a legislative mandate or public funding. Instead, ENCePP was designed to attract drug sponsors’ funding for pharmacovigilance research through ENCePP research centers [91]. State regulators in the past turned to the pharmaceutical association when drafting regulatory policy and referred to their industry counterpart as a partner, that extended to the oversight for post-market studies for example, where a representative of the EMA indicated,

*“We don’t commission studies ourselves… but again, the [European] Commission is in partnership with the industry associations…”* (EU1-EMA 2007).

A national commission in France recognized the need for independence from industry when its regulator was restructured, and assigned funds for independent pharmacovigilance research [88].

The Japanese regulator and industry associations were known to be enmeshed in formal and informal relational networks that tended “to blur the line between the private and the public realm” [92–94]. Health Canada’s *clientele pluralist* approach incorporated formal avenues for consultation and negotiation with industry to expand the range of policy options [95]. In the past, after receiving early notice of the agency’s thinking on issues, the industry association presented arguments that may have been accommodated and was invited to develop draft policy from which the agency worked [28, 96]. The agency’s limited resources led it to turn over some of its authority to private interests, fostering state-societal relations similar to that in Europe [28].

The FDA *pluralist* approach to policy development alternatively involves indirect involvement of private interests through judicial appeal [12, 13]. Consultation with industry takes place at the FDA’s discretion to refine regulatory systems, whose decision-making is based on managerial discretion. The social networks that arise from administrative interactions with product sponsors can, however, affect regulators’ perspectives and in turn policies that Carpenter [97] characterizes as *corrosive* given their deregulatory effect [98, 99]. Access to freedom of information laws and public interest groups’ ability to sue the FDA for the release of information led to greater transparency.^9^ The Federal Advisory Committee Act of 1972 makes the minutes of FDA advisory committee meetings publicly accessible.

*“…we’ve had some public meetings about that. We’ve put out concept papers…it not only gives you best practices but it helps with transparency as well, I think”* (US2-FDA 2010).

The FDA formerly made more information publicly accessible than other regulators, although not all clinically significant information was released [100]. The EMA has become more proactive in this regard and now releases the clinical study reports that contain almost all the safety and efficacy data that companies submit to attain market authorization for a new drug [101]. In Canada, Vanessa’s Law will enable Health Canada to release the same amount of information as the EMA once regulations are finalized. The ability of these agencies to balance a sponsor’s interest in timely product authorization while ensuring safety and efficacy standards are met is crucial [102] given the potential harm from adverse drug effects [103]. Critics suggest that all regulators’ independence is undermined by their growing reliance on industry user fees that create a ‘dual loyalty’ [1, 97, 104, 105] and warn that faster drug approval times with lower quality evidence have potential serious safety consequences [106].

#### Representation in regulatory decision-making

The groups represented in regulatory forums and their perspectives guide regulatory decision-making. Representation thus has implications for the network’s ability to develop regulatory approaches that reflect societal values to enhance their legitimacy [2, 8]. In neo-corporatist governance models, affected interests are internally represented within executive decision-making structures. Conversely, in pluralist models affected interests are externally represented and given rights to challenge decisions through “notice and comment” provisions and judicial review, allowing only indirect influence. In *clientele pluralist* models interests operate externally but with the active consent of the government.

In France, the UK and Japan *neo-corporatist* representation in regulatory policy discussions involves government and industry associations. The EMA, in contrast, formally works through six scientific committees, a Patients’ and Consumers’ Working Party and a Healthcare Professionals’ Working Party. Although these committees comment on policies and advise the EMA, in general they have not participated in or had access to decision-making committee minutes [107].^10^ The systematic involvement of consumer and healthcare professional interests in the daily operations of the agency is thus scarce [82]. The EMA *centralized* product review process consists of formal and informal interactions between two Member State rapporteurs (one nominated by the product sponsor) who oversee the evaluation and the industrial sponsor, that are insulated from public purview. The sponsor is assisted in developing its product by a scientific advice review group, comprised of members of the CHMP that leads to a preliminary agreement between the agency and the sponsor concerning the requirements for a successful application [82, 108]. In coordinating post-authorization and post-formulary listing decisions, France’s *Comit*é *de liaison* had wide representation that included drug benefit insurers whose goals led the committee to consider issues of real-world drug use and effects that prompted *active* surveillance [109].

In the US, the FDA also interacts with sponsors to help assure that products will meet the approval process requirements. Affected interests are otherwise externally represented in policy development with rights to challenge decisions through “notice and comment” provisions and judicial review, allowing indirect influence. In the post-market phase, the FDA engages diverse organizations to inform its decisions to commission pharmacosurveillance research through the Federal Partners Program [97, 104, 105].

*“The Drug Safety Board is essentially an advisory board to the (FDA) Center Director. And so it includes a lot of leaders…that are involved in the scientific review of regulated products…Veterans Administration, Department of Defense…the Agency for Healthcare Research and Quality…the National Institutes of Health”* (US2-FDA 2010).

Although FDA advisory committee members that evaluate the evidence base for product authorizations must declare conflicts of interest (COI), such self-disclosure has not necessarily led it to exclude scientists with conflicts from advisory committee votes.

In the Canadian *clientele pluralist* context, interests operate externally but with the active consent of government. Pharmaceutical trade associations have representation in Health Canada’s policy network. The Canadian academic policy community’s advocacy for a publicly funded center for pharmacosurveillance research made *post-market* assessment an area of contested governance. In response, the federal government launched the Canadian Drug Safety and Effectiveness Network through the Canadian Institutes of Health Research that commissions pharmacosurveillance research from academic centers. Although Health Canada currently has limited authority to impose safety studies on manufacturers, it has begun to engage DSEN [110].

**Fig. 2:**
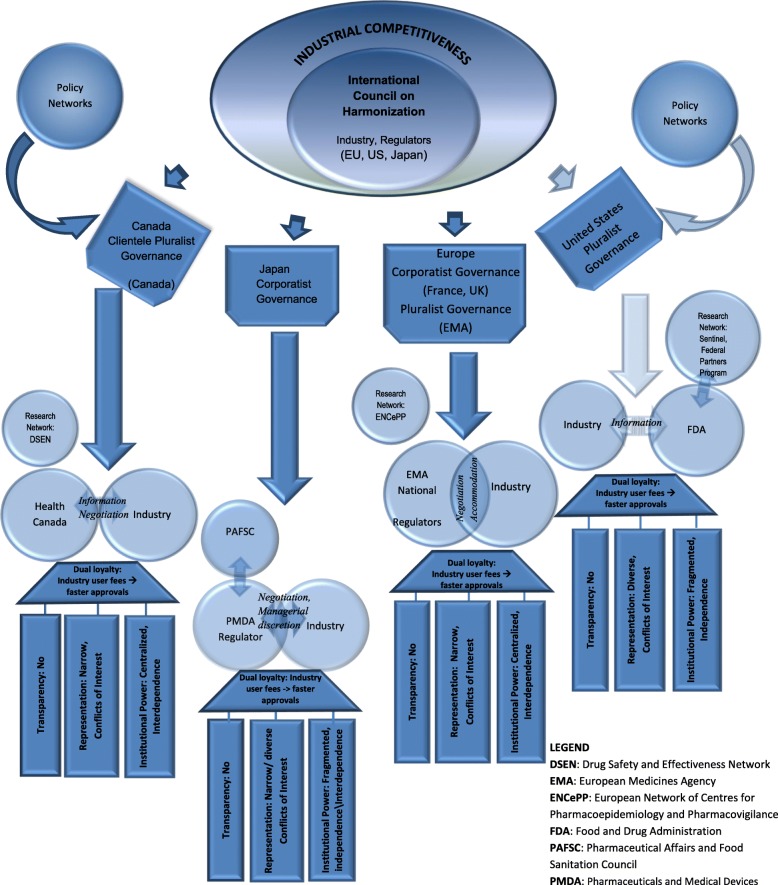
Global Harmonization Networks and Medicines Regulation

A history of collaboration between industry and government exists in Japan, where the government operates across public and private sectors. Regulatory policy is underpinned by a series of “linkages and privileged points of access and communication between government and industry, the effect of which is to integrate the industrial policy community and to facilitate…the formation and representation of interests” [92]. Despite a history of policy networks that insulated regulatory policy making from public debate [92, 94], the oversight and advice of the PAFSC to the Minister on regulatory standards and product reviews has introduced the voice of clinical and social scientists within the policy process [111] (Fig. 2).

Approaches to COI in regulatory agencies and the ICH presuppose and promote the idea that COIs cannot and need not be eliminated as the risk of bias can be managed. Representation in ICH decision-making is limited to regulators and industry trade associations, the latter of which prioritize commercial interests. The ICH excludes university-based experts (epidemiologists and pharmacologists), public drug benefit plans, health professional associations and consumer representatives whose experience in assessing drug therapies would lend insight and counteract industry members’ commercial bias [97, 112].^11^ Official statements about COI in laws and codes of practice in the EMA, MHRA, FDA, the French National Agency of Medicine and Health Products Safety (ANSM) and Health Canada suggest that these regulators have adopted strong policies to deal with COIs among experts and advisory committee members. In practice however, conflicts are managed, leaving open the very real possibility that COI could either influence their decision-making or appear to influence it [113, 114].

#### Decision-making approach

The processes involved in regulatory decision-making can be distinguished not only by government-industry relations that lean to varying degrees toward neo-corporatist or pluralist approaches; they also vary in the extent to which they are based on one of two competing approaches to decision-making: *analytic* and *cybernetic* that use different processes to organize problems to address uncertainty [115]. The *analytic* paradigm involves a comprehensive assessment of available data [116], while the *cybernetic* approach focuses on a limited range of critical variables [117]. The FDA and Japanese PMDA use a managerial discretion model of decision-making with an *analytic* lens; they were the only agencies that re-analyzed drug sponsors’ pre-market randomized controlled trial data for several decades. The FDA pools the data for drugs in the same class to increase the power to detect safety signals leading to the identification of adverse drug events not previously reported by industry [118]. A series of product market withdrawals due to serious or fatal adverse drug events led Congress to insist the FDA adopt an analytic approach. Successive congressional investigations of the FDA and the drug industry criticized FDA decisions and demanded tougher regulation and oversight by a competent authority [68].^12^

*“…in…Dec ‘09…the single, the sole recommendation of the GAO was that the commissioner, the head of the FDA develop a plan to transfer more of the responsibility to OSE (Office of Surveillance and Epidemiology)”* (US2-FDA 2010).

Guided by a *cybernetic* approach to decision-making, European and Canadian regulators primarily review summary data, even though there are instances where the UK MHRA issued regulatory warnings based on its review of sponsors’ clinical trial data [119]. The EMA required submission of clinical trial data as of 2011. Health Canada may request clinical trial data but exercises this authority rarely if at all. Decision-making within the ICH would appear to follow a cybernetic approach based on consultation with industry rather than considering more extensive expertise and perspectives in harmonizing regulatory standards.

#### Implications of litigation for regulation

As legal rulings supersede the authority of a regulatory agency, understanding the extent to which legal instruments are used to influence regulatory governance and policy for the ICH and member jurisdictions lends critical insight. Within member states, the degree to which litigation may offer a channel for consumers harmed by unsafe drugs to seek compensation from drug sponsors varies widely. Legal channels also have implications for the extent to which regulatory agencies and legislatures are informed of the burden unsafe drugs pose and may exert pressure on them for greater accountability. In the US, legal suits were found to fragment FDA authority and drive relative transparency [120] (Table 2). Class action and fraud lawsuits against product sponsors have, for example, hastened change in regulatory processes by contributing to the evidence available on product risks [120, 121]. Although the FDA attempted to consolidate its authority by asserting that its decisions should pre-empt nearly all legal action concerning drug safety, the Supreme Court ruled FDA authorization of a drug label does not pre-empt state law product liability claims that require drug makers to adequately warn about product risks [122]. In cases where industry conceals relevant information and the FDA lacks the capacity to uncover product harms, the legal system supplements agency oversight [123]. American class action lawsuits involving rofecoxib (Vioxx®) for example, led to penalties of $US5.3 billion for its manufacturer.^13^

In the UK, France, Japan and Canada, legal suits have not challenged regulatory decisions to the same extent, centralizing regulators’ authority. British and French laws make it difficult to launch class action lawsuits against pharmaceutical companies. In the UK, a Group Litigation Order allows multiple cases to be managed rather than submit a class action claim. A Group Litigation Order requires all claimants to bring individual claims that are registered and administered together. Financing individual claims in the UK has posed a barrier to the launch of lawsuits against pharmaceutical companies [124]. In France, the *Hamon* law (2014) deemed that class action suits may only address infringement of competition and must be launched by one of 15 approved national associations [125]. In Canada, class action lawsuits occur only occasionally [126]. Japan introduced class action legislation in 2016. The American judiciary has thus had the greatest effect on the regulatory process to date.^14^

## Discussion

Regulation is a distinctive form of policy-making and public control where the mechanisms and processes that guide the formation of regulatory standards meld political, scientific and technical dimensions [29, 127]. In advancing harmonization, transnational pharmacogovernance affords ICH members a means to leverage their interdependence to reshape international bargains including the potential to replicate their domestic rule structures through a transnational network with extensive global reach [31].

Co-regulatory governance guided the first quarter century of the ICH and orients its current decision-making [7, 45]. The success of the ICH in harmonizing pre-market regulatory standards fostered its acceptance by founding members. ICH governance involving bilateral negotiation parallels modes of state-societal relations and regulatory representation in Canada, France, the UK and Japan involving regular consultation with private members who co-develop norms and rules. The EMA is guided by Article 61 of the European Commission Regulation 726/2004 that specifies consensus-based negotiation guided by such *soft law* instruments as technical guidelines, parallel to ICH governance [84, 85]. With experience in harmonizing standards across the EU, the EMA held a “leadership role in promoting international regulatory cooperation” in the ICH [17]. ICH co-regulatory governance aligned with the European Commission [30, 85] and Member States, whose standards were accepted as ICH guidelines (Table 1).

European and ICH governance trajectories reveal parallels in the timing of transnational harmonization, regulatory initiatives (common technical document, risk management plans) and transparency reforms (Table 3). In France the inquiry into the AFSSAPS that highlighted the manner in which COI undermined regulatory decisions through the Mediator scandal hastened a drive for greater transparency to alleviate the appearance of and actual COI. In response, the practice of allowing a pharmaceutical trade association member to chair an ICH Working Group developing a regulatory guideline was curtailed. Parallel reforms shaped longer term governance and policy in the ICH and European regulators as well. In the ICH, industry financing was replaced by regulatory member contributions (the FDA offered $US500, 000 per year for 5 years from 2016). The US industry member noted, “The shift in the balance of power from regulated industry to regulators that the reforms will herald in is ‘appropriate’ and, if anything, the reforms should have been introduced sooner” [128]. Whether ICH governance reforms actually shift power dynamics remains to be seen as industry networks first comment on a new draft guideline and may frame it from their perspective, followed by a review by regulators. EMA’s push for transparency involved enabling the release of RCT data (that was legally contested by industry) and the potential for public hearings related to pharmacovigilance.

**Table 3:** ICH governance trajectory: Parallels to European regulator governance

Year	ICH	Europe
1987–95	Transnational harmonization Harmonization of pharmaceutical standards across EU, US and Japan through ICH • Consensus-based governance: among regulators and industry trade associations in EU, US and Japan; • Funded and housed by IFPMA; • Industry members may chair standards Working Groups; • Industry and regulators have equal votes in determining standards; • Guidelines on safety, efficacy and quality reflect EMA standards	Transnational harmonization Harmonization of standards pioneered in EMA; EC single market for pharmaceuticals demonstrates feasibility • Consensus-based governance: among member state regulators; • EU Centralized and decentralized process for authorizing medicines; • Centralized concertation process for innovative products, GMP, labelling, advertising guidelines, rules for blood products and vaccines; • UK and France: regulators use corporatist governance and negotiation with industry.
2000–1	Common technical document • Harmonized product dossier for electronic submissions	Common technical document • Used in EMA • EU Clinical trial directive
2004–5	Risk Management Plan Expands ICH role from harmonization to development of new standard	Risk Management Plan UK House of Commons Health Committee criticizes MHRA relationship with industry and routine policy consultations; advises review of MHRA, post-market surveillance
2010–12	Conflict of Interest addressed • Industry members can no longer chair an ICH Working Group developing an international standard	Conflict of Interest addressed • Inquiry into France’s AFSSAPS concerning Mediator (benfluorex) highlights COI undermined regulatory decisions;
2012–5	Governance reforms: • Negotiated governance: for decisions with no consensus, regulators vote; • ICH is legal entity under Swiss law; • Assembly; Management Committee; • Membership fees fund ICH; • Membership expands jurisdiction	Governance reforms: • New French regulator (ANSM) • EMA enables researcher access to clinical trial data • EMA possibility of public pharmacovigilance hearings

Sources: 50 Years, EU Pharmaceutical Regulation Milestones, European Commission, https://www.fda.gov/downloads/Drugs/NewsEvents/UCM500013.pdf, http://www.ema.europa.eu/ema/index.jsp?curl=pages/about_us/general/general_content_000628.jsp&mid=WC0b01ac058087addd

Interdependence between the EU and Japan would appear to have contributed to regulatory convergence on risk management plans. Endorsement of industry-led post-market studies is consistent with a neo-liberal trend that engenders greater reliance on industry given its market power [61, 129]; that may lead to perceptions that safety competes with harmonization [130, 131] as pharmaco-epidemiologists advise post-market studies be conducted independently, contrary to the current trend, to guard against conflict of interest*.*

Transnational guidelines, developed through negotiation with private sector actors that reduce sponsors’ burden in meeting regulatory standards are framed as market-based solutions advanced by experts that balance regulation with industrial competitiveness [15, 23], support investment in new products and enhance patient access [17, 132]. As a parallel transnational regulatory network that served as a pre-ICH consultation forum for risk management plans, CIOMS is similarly guided by the techno-expert model (where pharmaceutical industry technical experts lend authority). Techno-expert models attain legitimacy through transparency, public reporting and accountability, mechanisms that increase the likelihood that policy reflects the values of democratic legislatures. The legitimacy of the techno-expert model may diminish when regulatory decisions lack public acceptance, particularly given the limited channels for incorporating societal perspectives into regulatory decisions imbued with scientific uncertainty [2, 29].

Despite growing international convergence on supranational standards that become de facto global standards as the ICH and WHO foster their adoption internationally, states retain a level of autonomy [35]. A more independent American approach to pharmacovigilance and different adverse drug event reporting standards coincided with the confluence of more extensive legislative oversight of the FDA, broader consultation with related healthcare agencies, and a culture of litigation whose cumulative effects may recalibrate risk perception and management to foster improvisation in regulatory standards. An alternate governance process in Japan with independent oversight of the regulator by the PAFSC led the Ministry to adopt a more anticipatory approach to adverse drug events. Inclusion of an independent oversight committee for the regulator such as PAFSC, combined with the managerial discretion-guided decision making within the regulator, was found to counterbalance neo-corporatist relations (Fig. 2).

## Conclusion

In a globalized context, transnational networks increasingly replace state governance processes, where transnational sectoral autonomy dominates [21, 24, 133]. This migration of authority from state to supranational networks may shift relations of power that prompt questions of legitimacy [22]. The capacity of transnational pharmacogovernance networks to craft policy in the public interest depends on the governance process that guides the formation of formal decision rules, the incentives of the actors involved and the types of policy issues addressed. Networks’ incentives to form policies in the public interest are highest when governments impose a level of oversight that prompts accountability [134].

Representation in the transnational network’s governance framework has implications for its ability to develop standards that reflect societal interests [2]. Although medicines regulation is considered a technical non-discretionary matter, the latitude of ICH members to determine areas for harmonization and the uncertainty inherent in new product reviews [97] suggest more extensive academic research-based expertise and representation may enhance policy capacity in the public interest. A precautionary principle to COI which in practice means that rather than managing COI it should be avoided, would improve transnational and national regulatory processes.

As transnational networks become adept at responding to emerging political challenges through harmonized standards that level the international playing field, they contribute to a multinucleated global system comprised of a series of sectoral networks, that create circuits of power organized around issue areas [23]. As interdependence shapes transnational pharmacogovernance, the parallels found between ICH and member state governance, including the nature of representation and decision-making that guides ICH standard setting, reflect emergent patterns (Tables 1, 2 and 3). Although such parallels suggest a thread through which interdependence may shape transnational governance, admittedly, the factors that affect the design of transnational regulatory governance and how they contribute to regulatory convergence necessitates further analysis. Areas of policy dissonance that could be further explored in which the ICH has not been officially involved include:

1. Accelerated drug authorizations: Although the EMA’s ‘adaptive pathways’ converge with the US and Canada’s policies for accelerated authorizations, Japan applies this approach only to regenerative therapies such as stem cells. Allowing new therapies to enter the market though accelerated authorizations without Phase II or III clinical trials reflects regulatory improvisation that has hastened controversy [106].
2. Dissonance in transparency: Although regulators have enhanced access to clinical trial data, adverse event databases and rationales for product refusals and acceptances, differences among agencies persist. Even though the FDA enables access to advisory committee assessment reports, while the EMA does not make CHMP proceedings available, the quality of FDA advisory committees has declined as experts often have COI; the appropriate experts may be missing or must limit their comments; and not all drugs are discussed by an advisory committee. The EMA has been found to communicate more with sponsors during the drug review phase than the FDA, such that sponsors’ applications are at times withdrawn before being rejected by the EMA [135].
3. EMA-member state regulator decision-making dissonance: The European system of mutual recognition was designed to facilitate simultaneous marketing authorization across member states, once a sponsor attained market authorization from one regulator. EU member states were initially reluctant to accept the decisions of other agencies, objecting to all but one of the 300 applications submitted from 1975 to 1995 [136], reflecting the complexity of establishing legitimacy and trust in institutional relations. As institutional processes and relations improved, the EMA system of mutual recognition became the EU system of regulatory approval.
4. EU pharmacosurveillance institutional dissonance: Issues may arise where one agency depends on others for information, especially if the work of different agencies is perceived to be of a different quality as sometimes occurs. Different levels of involvement by supranational and national regulators can also result in unnecessary duplication of work, that can arise at times when communication is sub-optimal among the EMA and member states [137].

In elucidating how similar variables intersect in different national contexts, such analyses may better clarify the conditions that foster policy convergence in the public interest.

While globalization and the ascendance of transnational networks may cast the state as superfluous, state capacity to determine the manner in which soft laws and technical guidelines are articulated offers a counter balance [35]. Just as an integral element of a jazz standard is the unresolved texture a dominant seventh chord introduces before its resolution, transnational policy dissonance may be integral to a resolution that enhances harmonized standards, suggesting a means for globalization to advance public policy.

### Limitations and future research

While we are confident about our insights, we recognize that there were limitations in our methodology that may have limited our conclusions about the intersection between transnational and national/supranational standards and the multiple possible nuances in the ways that national or regional decisions interact with harmonization efforts that we have not addressed, as highlighted above. Although we gathered perspectives of members of the pharmaceutical industry from sources in the literature, they were not included in the interviews and that could be considered a limitation. Future research could address this deficiency by incorporating the perspectives of, among others, industry, healthcare professionals and consumers to further explore areas of dissonance. Although both documentation and interviews were used to arrive at our insights there were areas that were only covered by one type of source and therefore we could not conclusively determine their completeness and accuracy. Discerning patterns in transnational and national governance approaches and the contextual factors that affect their emergence remains relevant for future research concerning regulatory convergence.

## Additional file

Additional file 1: **Appendix 1.** Key Informant Interviews Codebook. **Appendix 2.** Quotations from Key Informant Interviews and source documents. (DOC 63 kb)

## Abbreviations

ABPI: Association of the British Pharmaceutical Industry
AFSSAPS: French Agency for the Safety of Health Products
ANSM: French National Agency of Medicine and Health Products Safety
CHMP: Committee for Human Medicinal Products
CIOMS: Council for International Organizations of Medical Sciences
DSEN: Drug Safety and Effectiveness Network
EMA: European Medicines Agency
ENCePP: European Network of Centres for Pharmacoepidemiology and Pharmacovigilance
EPPV: Early Postmarketing Phase Vigilance
FDA: Food and Drug Administration
FDAAA: FDA Amendments Act
GPSP: Good Postmarketing Study Practice
GVP: Good Vigilance Practice
ICH: International Council for Harmonization of Technical Requirements for Registration of Pharmaceuticals for Human Use
MHLW: Ministry of Health, Labour and Welfare in Japan
MHRA: UK Medicines and Healthcare products Regulatory Agency
NICE: UK National Institute for Health and Care Excellence
PAFSC: Pharmaceutical Affairs and Food Sanitation Council in Japan
PAL: Pharmaceutical Affairs Law
PBRER: Periodic Benefit Risk Evaluation Report in Japan
PMDA: Pharmaceuticals and Medical Devices Agency in Japan
PSUR: Periodic Safety Update Report

## Footnotes

1 The areas where the ICH develops harmonized standards include: quality guidelines (e.g., conduct of stability studies), efficacy guidelines (e.g., design, conduct, safety and reporting of clinical trials), safety guidelines (uncover potential risks such as cardio-toxicity) and multidisciplinary guidelines (e.g., the ICH medical terminology). See: http://www.ich.org/products/guidelines.html. Accessed 3 Aug 2018.

2 Each member forms a contact network of experts within their organization from which their representative is drawn to ensure Expert Working Group discussions reflect members’ views and policies.

3 For example, the US took action on biological product safety issues three times more frequently than the EU from 1995 to 2007 [58].

4 Related initiatives of the common committee included enhanced quality assurance systems, Eudra Vigilance (a European-wide electronic ADR database), transparent communications on product safety, coordinated resources for pharmacoepidemiologic studies and advancing research methods in the field of pharmacovigilance.

5 The FDA attained the authority to require manufacturers to undertake Risk Evaluation and Mitigation Strategies (REMS) and develop Risk MAPs that exclude *active* surveillance. REMs and RiskMAPs consist of updated medication guides, healthcare provider education to support safer use, risk communication strategies, a prescriber agreement and a patient-physician agreement [138].

6 The PAFSC is comprised of 16 committees and 21 subcommittees. The PAFSC members include experts in such fields as medicine, pharmacy, biology, dentistry, nursing and social science based in universities, hospitals and research institutes; there are 55 permanent members and about 400 temporary members. Areas addressed by the PAFSC include the determination of regulatory standards for drugs, review of new applications, re-examination and re-evaluation drug applications, and judgments concerning relief funds payment under the provisions of the ADR Relief and Research Promotion Fund Law [80].

7 The British Government’s 1987 review of the agency, prompted by industry’s complaints of slow reviews, was co-chaired by a former ABPI chair and led to several changes including industry fees to fund the new product authorization process. The government also agreed to consult industry on the agency’s management performance [94].

8 The government ignored most of the Committee’s recommendations [95].

9 The FDA’s relative transparency reflects its adherence to the Administrative Procedures Act (1946) and the Freedom of Information Act (1967) that require agencies to record how decisions have been made, and in the case of pharmaceuticals to make those records public following marketing authorization.

10 A pilot project in 2016 involved patients in the Committee for Human Medicinal Products (CHMP) benefit-risk evaluation of medicines meetings. Patient representatives are in some cases funded by pharmaceutical companies [113]. In contrast, consumers are users of health services overall who may be more objective [114].

11 Individuals are not eligible to become members or observers of the ICH. Articles of Association 17 and 18 allow the ICH Management Committee to invite organizations as ad hoc observers to the Assembly, while Article 19 makes provisions for the termination of observer status [139].

12 Although the FDA approach to pharmacovigilance entails greater independence from industry, the FDA has not always acted on the safety signals detected. In the case of rosiglitazone (Avandia®) for example, while the FDA had internal analyses of an increase in cardiac events as an adverse drug effect, it did not issue a safety warning until an independent study was used to criticize the FDA for not acting on the information [140].

13 Patients harmed by rofecoxib (Vioxx®) filed a class-action lawsuit against Merck. The company settled the product-liability lawsuits for $US4.85 billion. Related litigation, including a multi-district class action lawsuit against Merck in New Jersey federal court alleged it concealed safety information from company-sponsored trials from investors. Merck settled the lawsuit by paying $US830 million to investors who bought Merck stock [141].

14 The EU developed a regulation mandating that Clinical Study Reports be published following marketing authorization and launched a proactive publication policy (Policy 0070) to upload clinical reports submitted for marketing authorizations on a publicly available website in 2014. Despite legal action by companies against the EU to annul the EMA’s decision to release clinical trial data, the policy stands. The clinical data are available to download and re-use by academics and for non-commercial research. Commercially confidential information may however be redacted before the data are released [132].
