# Supplementary material for: Transnational pharmacogovernance: emergent patterns in the jazz of pharmaceutical policy convergence
Source: Global Health. 2018 Aug 22;14:86. doi: 10.1186/s12992-018-0402-5 (PMC6106922; doi:10.1186/s12992-018-0402-5)
Supplement: Supplementary file 1 — Appendix 1. Key Informant Interviews Codebook. Appendix 2. Quotations from Key Informant Interviews and source documents. (DOC 63 kb) [file 12992_2018_402_MOESM1_ESM.doc]

**Appendix 1**

**Key Informant Interviews Codebook**

| Codes | Definition | Quotes |
| --- | --- | --- |
| *Clientele* pluralism | Regulator’s primary contact group is industry; regulator occasionally takes independent stance | *“Under the NOC/c policy, the sponsor plays a part in the decision concerning the type of post-market study to be conducted as they must undertake to design, carry out and report on the post-market studies.”* - Health Canada written response, 2010 |
| Collaborative | Joins forces in a formal or non-structured partnership for the purpose of pharmacovigilance. | *“…we work in a collaborative model for anything we do have to do here at the Center for Drugs…generally OSE and OND staff form a safety issue team, so if the safety issue comes up they form a team, they develop a work plan, etc. and each goes and does thier respective piece based on their area of expertise. And then they, you know, meet frequently, are talking with each other etc. and to discuss their findings, conclusions, and their recommendations.”*- US2-FDA, 2010 |
| Consultative | Engages external advisors in pharmacovigilance decision-making. | *“The Drug Safety Board is essentially an advisory board to the Center [CDER] director. And so it includes a lot of leaders of the organization in the Center for Drugs that are involved in the scientific review of regulated products. It does have members from outside the FDA but they’re all government members…[including] Veteran’s Administration, Department of Defense, both of which have big healthcare systems …the Agency for Healthcare Research and Quality, which is another federal agency, the National Institutes of Health and perhaps some others. The board meets basically a monthly basis and it looks at the specific safety issues, it also reviews how CDER…is communicating these messages as well…”*- US2-FDA, 2010 |
| Corporatism | Interests are *internally* represented within executive decision-making structures | *“We are no longer doing the routine five-year renewal. We used to do it in the past, but what has happened with it is that it became more like an administrative procedure.*” - EU1-EMA, 2007  *“We are also clarifying the leading role of the regulators compared to that of the industry [because] from the European perspective where we have in the past years been under increased scrutiny over, you know relations with the industry.”* - EU3-EMA, 2015 |
| Pharmacogovernance | The manner in which governing structures, policy instruments and institutional authority that enable the development, implementation and enforcement of policies and processes are managed to promote societal interests including protection of public safety | *“So even if in the future we will have both members and observers, but even as an observer you can of course participate fully in the discussion and all that. Although, you do not necessarily participate in the decision making.”* - EU3-EMA, 2015  *“Our regulations don’t contemplate a…or require a pharmacovigilance plan so we think the guideline is a good guideline it’s just that we don’t have a regulatory mechanism for it.”*- US4-FDA, 2015 |
| Regulatory  Independence | Independence was defined as not depending on another's authority or resources for support. | *“When we were preparing for our pharmacovigilance legislation, we felt for example we thought it would be better to first to see the legislation come through, and then analyze you know how the ICH guidelines fit into this legislative framework because obviously legislation has priority over guidelines.”* –EU3-EMA, 2015    *“I think it is more the semantics because clearly they say different things. An adverse event as we define it does not have to have a suspicion of causality whereas the ICH standard says only if suspected [it] is related to a medicine. We think that casting it broadly like that is the best approach for seeing things that may be you might not see otherwise*.*”* – US4-FDA, 2015  *“Our regulations don’t contemplate a…or require a pharmacovigilance plan so we think the guideline is a good guideline it’s just that we don’t have a regulatory mechanism for it.”* – US4-FDA, 2015 |
| Regulatory  Interdependence | The mutual dependency of state interests. Can be a positive force for cooperation, as nations come to accept and benefit from that relationship. | *“In the EU we recently introduced very important, very important overhauls of our pharmacovigilance system, which obviously was a strong incentive for us to bring pharmacovigilance to ICH for discussion because we had the old guideline which did not – no longer synced with our new regulatory system.”*- EU3-EMA, 2015  *“ICH guidelines become FDA guidance documents once they reach Step 4 and are approved (“signed off”) by the regulatory authorities in ICH. If an FDA Guidance to Industry already exists on a particular topic, it is retired upon publication of the new ICH/FDA guidance*.” – US5-FDA, 2015 |
| Transparency | Decisions, decision-making mechanisms, policies and records are open to public scrutiny and are understandable to lay public in addition to scientific community. | *“if you put your best practices out there it just makes you transparent…If people can’t follow, can’t understand what you did or you tell them what you did- you just make a decision, that’s not a good thing.”*- -US2-FDA, 2010 |

**Appendix 2**

**Quotations from Key Informant Interviews and source documents**

| **Key Informant**  **Jurisdiction** | **Date** | **Topic** | **Quote** |
| --- | --- | --- | --- |
| EU3-EMA | August 2, 2015 | ICH guidelines | *“In the EU we recently introduced very important, very important overhauls of our pharmacovigilance system, which obviously was a strong incentive for us to bring pharmacovigilance to ICH for discussion because we had the old guideline which did not – no longer synced with our new regulatory system.”* |
| EU3-EMA | August 2, 2015 | Pharmacovigilance legislation | *“When we were preparing for our pharmacovigilance legislation, we felt for example we thought it would be better to first to see the legislation come through, and then analyze you know how the ICH guidelines fit into this legislative framework because obviously legislation has priority over guidelines.”* |
| EU3-EMA | August 2, 2015 | ICH decision-making (Representation) | *“So even if in the future we will have both members and observers, but even as an observer you can of course participate fully in the discussion and all that. Although, you do not necessarily participate in the decision making.”* |
| EU1-EMA | June 20, 2007 | Risk management plan | *“If we think it’s important enough to put what we call a ‘specific obligation,’ then it has to be followed completely. The only thing is, we don’t use that measure - we use it to a certain extent - but we are more likely to use what we call a ‘follow up measure’ where the company makes an undertaking…the committee has to endorse it, and the company has to follow it.”* |
| EU1-EMA | June 20, 2007 | Risk management plan | *“If it is not followed, then we can have - we can take measures. For a lot of the studies, for instance, if there is a question about the feasibility of the study, or if there is some uncertainty, then we opt for a follow-up measure, because that one gives us more flexibility on both sides. We are doing this, because there is bit of uncertainty at the moment about the introduction of the new tools, you know the risk management plan, the studies that are linked to it. Because it is an early phase, we tend to take, if you like, the - we go along more with these follow-up measures.*” |
| EU2-EMA | March 12, 2010 | Risk management plan | *“What we found is that sometimes these studies are either not carried out because companies say that it is not possible to carry out these studies that have been requested in the EU.”* |
| EU3-EMA | August 2, 2015 | Industry-Regulatory Relationship | *“We are also clarifying the leading role of the regulators compared to that of the industry [because]from the European perspective where we have in the past years under increased scrutiny over, you know relations with the industry.”* |
| EU2-EMA | March 12, 2010 | Research network | *“ENCePP is mainly about capacity building …we realize that some of these studies weren’t carried out, some of them were carried out in the US because companies said it was difficult for them to carry them out in Europe…so it’s also to promote this type of research being undertaken in the EU.”* |
| US3-FDA | March 19, 2010 | Research network | *“The FDA needs these questions answered and we can’t do it ourselves, so we do it in collaboration with outside groups that have both the data and the expertise.”* |
| US3-FDA | March 19, 2010 | Research network | *“We do have outside contractors who work on epidemiology studies with us but we are paying them through a public competition…we’ll review these applications and select the best one. And they can work together, like the study on attention deficit hyperactivity disorder is across multiple contractors and they figure out how to work together…”* |
| US5-FDA | 2015 | ICH guidelines | *“ICH guidelines become FDA guidance documents once they reach Step 4 and are approved (“signed off”) by the regulatory authorities in ICH. If an FDA Guidance to Industry already exists on a particular topic, it is retired upon publication of the new ICH/FDA guidance.”* - FDA, 2016 |
| US4- FDA | 2015 | ADR reporting | *“I think it is more the semantics because clearly they say different things. An adverse event as we define it does not have to have a suspicion of causality whereas the ICH standard says only if suspected [it] is related to a medicine. We think that casting it broadly like that is the best approach for seeing things that may be you might not see otherwise.”* |
| US4-FDA | 2015 | Policy, law and Regulation | *“Our regulations don’t contemplate a…or require a pharmacovigilance plan so we think the guideline is a good guideline it’s just that we don’t have a regulatory mechanism for it.”* |
| US3-FDA | March 19, 2010 | Research methods | A priority is to develop “*better epidemiologic methods to handle confounding in observational data*”. |
| US3-FDA | March 19, 2010 | Research methods | *“We envision, especially with the FDAAA requirements … that we can require these observational studies, that firms will be doing this more and more, and so that is why the best practices guidance.*” |
| France- Inspection Générale des Affaires Sociales Report | January 21, 2011 | Benfluorex-delay market withdrawal | The AFFSaPS was “*…structurally and culturally in a situation of conflict of interest...by a sort of institutionalised cooperation with the pharmaceutical industry that leads to a co-production of expertise and of the decisions that stem from it*”.  <http://english.prescrire.org/en/81/168/46752/0/NewsDetails.aspx> |
|  |  |  |  |
| EU1-EMA | June 20, 2007 | Market renewal | *“We are no longer doing the routine five-year renewal. We used to do it in the past, but what has happened with it is that it became more like an administrative procedure.*”  *“It’s the other – with our new registration, it’s the other way around. In other words, the default will be that they will get an extension for forever, unless the regulator for the – basically, our legislation has changed in emphasis. It’s also the company had to justify a positive benefit risk to get another five years; the law has changed whereby the company will get that open ended authorization, unless the regulator can say that there is an ongoing safety issue for which we need another five year period. Am I clear on that emphasis of the legislation? Because, what we had in the past is that the company has to justify – what we have now is the regulator has to justify that there is a safety issue significant enough to need another five year period.”* |
